# Draft Genome Sequence of *Mycobacterium avium* 11

**DOI:** 10.1128/genomeA.00766-17

**Published:** 2017-08-10

**Authors:** Michelle Yee, David Klinzing, Jun-Rong Wei, Martin Gengenbacher, Eric J. Rubin, Jung-Yien Chien, Po-Ren Hsueh, Thomas Dick

**Affiliations:** a Department of Microbiology and Immunology, Yong Loo Lin School of Medicine, National University of Singapore, Singapore, Singapore; b AITbiotech Singapore, Singapore, Singapore; c Department of Immunology and Infectious Diseases, Harvard T. H. Chan School of Public Health, Boston, Massachusetts, USA; d Department of Microbiology and Immunobiology, Harvard Medical School, Boston, Massachusetts, USA; e Public Health Research Institute, New Jersey Medical School, Rutgers, The State University of New Jersey, Newark, New Jersey, USA; f Department of Internal Medicine, National Taiwan University Hospital, National Taiwan University College of Medicine, Taipei, Taiwan; g Department of Laboratory Medicine, National Taiwan University Hospital, National Taiwan University College of Medicine, Taipei, Taiwan

## Abstract

*Mycobacterium avium* accounts for most lung disease caused by nontuberculous mycobacteria (NTM). The lack of effective chemotherapy calls for the discovery of new drugs. Here, we report the draft genome sequence of *M. avium* 11, a clinical isolate used as a screening strain for NTM-focused drug discovery.

## GENOME ANNOUNCEMENT

The slow-growing nontuberculous mycobacterium (NTM) *Mycobacterium avium* represents a complex (*M. avium* complex [MAC]) of at least four subspecies. Members of the MAC are opportunistic pathogens ubiquitous in nature. The organisms have been isolated from soil and water samples and infect a diverse range of hosts, including birds, swine, ruminants, and humans (1). MAC pathogens cause the majority of all NTM infections in the United States and other countries, with *M. avium* subsp. *hominissuis* being of high clinical significance (1, 2). *M. avium* subsp. *hominissuis* can cause lymphadenitis, pulmonary and soft tissue infections, as well as disseminated disease (2, 3). The absence of effective multidrug regimens renders treatment of MAC infections difficult (2). Disseminated infections are commonly observed in patients with AIDS and are very challenging to cure due to adverse drug effects and drug–drug interaction with background HIV therapy (2, 4). Hence, there is an urgent need for new effective drugs with minimal side effects and no drug–drug interaction. Here, we report the draft genome sequence of *M. avium* 11, which is used as a screening strain in our ongoing NTM-focused drug discovery program (5–7).

*M. avium* 11 was isolated from the bone marrow of an AIDS patient with disseminated MAC infection, including pulmonary infection (National Taiwan University Hospital, Taipei, Taiwan). This clinical isolate forms smooth, opaque, and domed colonies (3) on Middlebrook 7H11 agar after 3 weeks of incubation at 37°C. *M. avium* 11 is classified as *M. avium* subsp. *hominissuis* based on the 3′ region of the *hsp65* gene sequence (8) that shows 100% identity when aligned with the *hsp65* 3′ region of *M. avium* subsp. *hominissuis* 104 (GenBank accession no. NC_008595).

Genomic DNA of *M. avium* 11 was extracted from a log-phase liquid culture using the phenol-chloroform method as described previously (9). The DNA libraries were prepared using Covaris shearing and Illumina TruSeq Nano DNA library preparation kits. Two sequencing runs of 2 × 300-bp read lengths were performed using an Illumina MiSeq platform, generating a total of 8,879,671 paired-end reads (AITbiotech, Singapore). These paired-end reads were quality checked using FastQC and subsequently quality trimmed using fqtrim (https://ccb.jhu.edu/software/fqtrim) with a window size of 7, a minimum average *Q* score of 28, and a minimum posttrim length of 35. The resulting 8,386,266 paired-end reads after trimming were *de novo* assembled using SPAdes version 3.6.2 (10) (*k*-mer sizes of 33, 55, 77, 99, and 127), producing 70 contigs with an *N*_50_ contig size of 216,071 bp. This assembled draft genome is 5,448,889 bp in size with a GC content of 68.99%. Annotations were performed using the Rapid Annotations using Subsystems Technology (RAST) server version 2 (11) predicted a total of 5,141 coding sequences and 49 RNAs, which includes 46 tRNAs and 3 rRNAs. Interestingly, PHASTER (12) predicted two prophage regions in the genome, including one partial prophage containing 68 coding sequences (65.56% GC content) and one intact prophage containing 56 coding sequences (66.74% GC content).

### Accession number(s).

This whole-genome shotgun project has been deposited at DDBJ/ENA/GenBank under the accession number NISH00000000. The version described in this paper is the first version, NISH01000000.
